# Immunogenic Cell Death Induction by Ionizing Radiation

**DOI:** 10.3389/fimmu.2021.705361

**Published:** 2021-08-20

**Authors:** Mengqin Zhu, Mengdie Yang, Jiajia Zhang, Yuzhen Yin, Xin Fan, Yu Zhang, Shanshan Qin, Han Zhang, Fei Yu

**Affiliations:** ^1^Department of Nuclear Medicine, Shanghai Tenth People’s Hospital, Tongji University School of Medicine, Shanghai, China; ^2^Institute of Nuclear Medicine, Tongji University School of Medicine, Shanghai, China

**Keywords:** immunogenic cell death, ionizing radiation, ferroptosis, necroptosis, damage-associated molecular patterns, nanoparticles, hyperthermia, chemotherapy

## Abstract

Immunogenic cell death (ICD) is a form of regulated cell death (RCD) induced by various stresses and produces antitumor immunity *via* damage-associated molecular patterns (DAMPs) release or exposure, mainly including high mobility group box 1 (HMGB1), calreticulin (CRT), adenosine triphosphate (ATP), and heat shock proteins (HSPs). Emerging evidence has suggested that ionizing radiation (IR) can induce ICD, and the dose, type, and fractionation of irradiation influence the induction of ICD. At present, IR-induced ICD is mainly verified *in vitro* in mice and there is few clinical evidence about it. To boost the induction of ICD by IR, some strategies have shown synergy with IR to enhance antitumor immune response, such as hyperthermia, nanoparticles, and chemotherapy. In this review, we focus on the molecular mechanisms of ICD, ICD-promoting factors associated with irradiation, the clinical evidence of ICD, and immunogenic forms of cell death. Finally, we summarize various methods of improving ICD induced by IR.

## Introduction

As a major modality in clinical cancer treatment, radiotherapy (RT) cures or palliates cancer in more than 50% of patients (1). However, radiation resistance of some cancers remains a clinical problem (2). Therefore, it is urgent to find effective ways to solve this problem. RT utilizes ionizing radiation (IR) to induce cell death directly through damaging double-strand DNA (dsDNA) (1). Moreover, IR has the potential to produce antitumor immunity, which is suggested by the discovery of the abscopal effect (3). The abscopal effect refers to the retarded growth of distant metastases following irradiation of the primary tumor (4). IR drives an antitumor immune response through a series of mechanisms [such as the upregulation of major histocompatibility complex (MHC) class I molecules, intercellular adhesion molecule-1, and factor-related apoptosis (Fas)], one of which is the induction of immunogenic cell death (ICD) (5).

ICD is a type of regulated cell death (RCD) driven by cellular stressors, including chemotherapy, IR, targeted anticancer agents, photodynamic therapy, and high hydrostatic pressure. It initiates CD8^+^ T cell-mediated adaptive immune response through damage-associated molecular patterns (DAMPs) emission (6–8). The induction of ICD by IR depends on the type of radiation, radiation dose, fractionation schedule, and tumor types. In addition, ICD such as necroptosis and ferroptosis can improve the radiosensitivity of tumor cells (9, 10). Thus, ICD inducers might be an auxiliary treatment method to produce synergistic antitumor effect combined with RT. In recent years, immunotherapy has been a hotspot in cancer treatment and ICD activation might be a promising cancer therapy modality.

Herein, we mainly discuss the mechanism of IR-driven ICD, factors related IR-induced ICD, and clinical evidence. Lastly, we summarize the methods of enhancing IR-induced ICD.

## The Mechanism of IR-Driven ICD

The induction of ICD requires three factors:

1. Inducible damage-associated molecular patterns (iDAMPs): cytokines [such as interleukin-6 (IL-6)] and chemokines [such as C-X-C motif chemokine ligand 1 (CXCL1), CXCL2, CXCL10, and CC chemokine receptor 2];
2. Tumor-associated or tumor-specific antigens;
3. Constitutive damage-associated molecular patterns (cDAMPs): high mobility group box 1 (HMGB1), calreticulin (CRT), adenosine triphosphate (ATP), heat shock proteins (HSPs), and so on (11).

**Figure 1:**
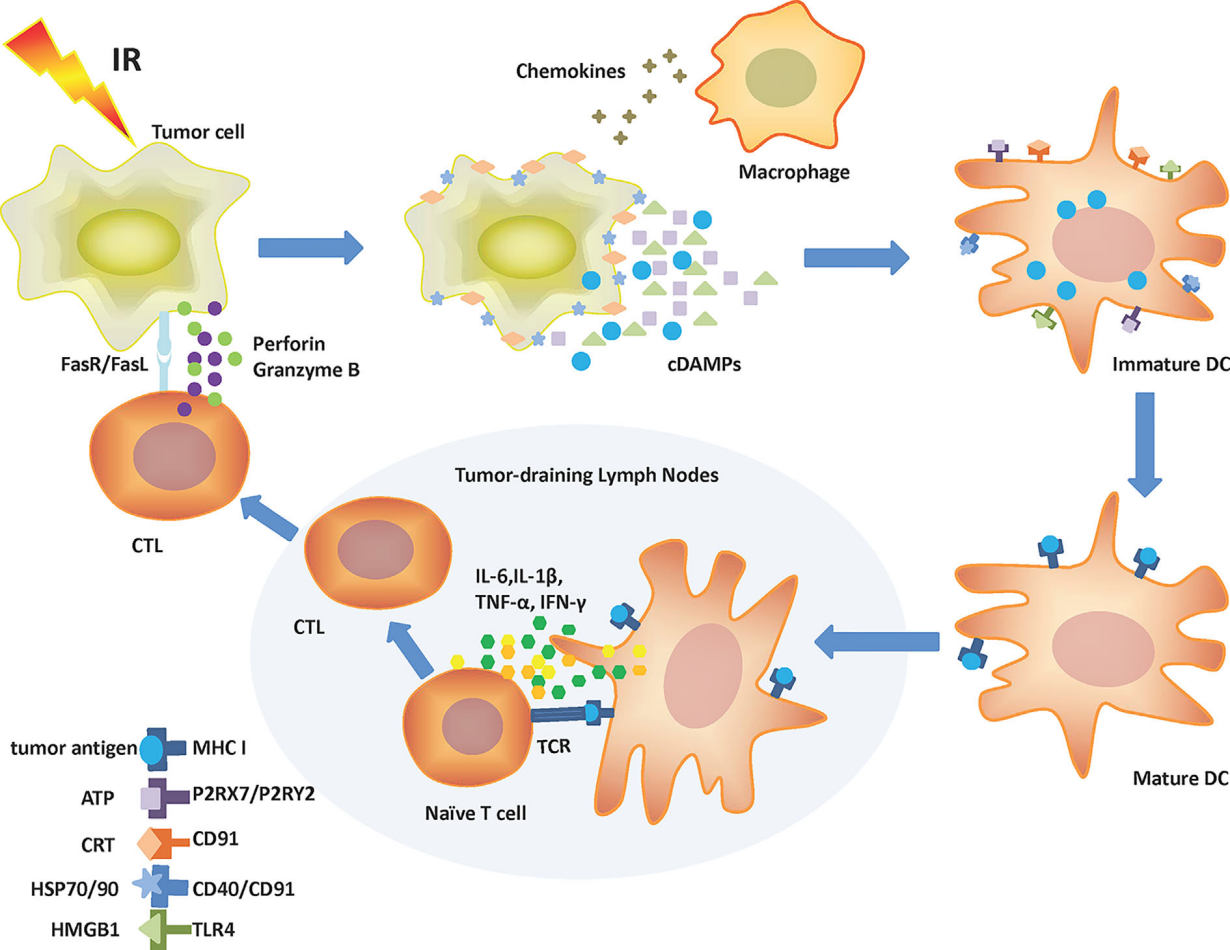
Mechanism of IR-driven ICD. Tumor cells release cDAMPs, chemokines, and tumor antigens following IR. Chemokines attract macrophages and immature DCs to tumor cells. cDAMPs like ATP, CRT, HSP70/90, and HMGB1 bind to corresponding receptors (P2RX7/P2RY2, CD91, CD40/CD91, and TLR4, respectively) on DCs leading to their activation. Recruited DCs can engulf and process tumor antigens. Mature DCs present tumor antigens to surface MHC-I molecules and then migrate to tumor-draining lymph nodes. There, DCs produce IL-6, IL-1β, TNF-α, and IFN-γ and cross-present antigens to boost T-cell differentiation to CTLs. CTLs can migrate to the tumor site and kill malignant cells by releasing perforin, granzyme B, or through the stimulation of the Fas/FasL pathway. ATP, Adenosine triphosphate; cDAMPs, Constitutive damage-associated molecular patterns; CRT, Calreticulin; CTL, Cytotoxic T lymphocyte; DC, Dendritic cell; Fas, Factor-related apoptosis; FasL, Factor-related apoptosis ligand; HMGB1, High mobility group box 1; HSP, Heat shock protein; ICD, Immunogenic cell death; IFN-γ, Type γ interferon; IL-1β, Interleukin-1β; IL-6, Interleukin-6; IR, Ionizing radiation; MHC I, Major histocompatibility complex class I; P2RX7, P2X purinoceptor 7; P2RY2, Purinergic receptor P2Y2; TCR, T-cell receptor; TLR4, Toll-like receptor 4; TNF-α, Tumor necrosis factor-α.

Irradiating tumor cells result in reactive oxygen species (ROS) production and collateral endoplasmic reticulum (ER) stress effects, which are required for cDAMPs release or exposure (12, 13). Specifically, cDAMPs involve HMGB1 and ATP release, CRT relocation, and HSPs exposure (14). cDAMPs act as “find me” signals (ATP and HMGB1) or “eat me” signals (HSPs and CRT) to mobilize antigen-presenting cells (APCs) such as dendritic cells (DCs), macrophages, and their precursors to tumor cells with the help of chemokines (15). The activation and maturity of APCs especially DCs depend on the interaction of cDAMPs and pattern recognition receptors (PRRs) on these cells. PRRs include P2RX7 (P2X purinoceptor 7), P2RY2 (purinergic receptor P2Y2), CD91, CD40, and TLR4 (toll-like receptor 4) (16). Belonging to the ionotropic purinergic P2X subfamily, P2RX7 is a 595aa protein expressed on almost all immune cells and promotes IL-18 and IL-1β secretion *via* the NLRP3/ASC/caspase-1 (NLRP3: NOD-like receptor protein 3; ASC: apoptosis-associated speck-like protein containing a caspase recruit domain) pathway when combined with ATP (17, 18). Interaction of ATP with P2RY2 promotes the recruitment of immature DCs, monocytes or macrophages, and neutrophils (6). CD91, also known as low-density lipoprotein receptor-related protein 1, is a endocytic and cell signaling receptor expressed on the surface of both normal and tumor cells (19). The CRT–CD91 interaction leads to the release of TNF-α (tumor necrosis factor-α) and IL-6 (20). CD91 also combines with HSP90 to facilitate cross-presentation (21). CD40 is a costimulatory molecule belonging to the TNF receptor superfamily and mainly expressed on APCs (22). HSP70 binding to CD40 results in CD8^+^ cytotoxic T-cell activation (6). As part of the TLR family, TLR4 is mostly expressed on the surface of innate immune cells and recognizes pathogen-associated molecular patterns like lipopolysaccharide and cDAMPs like HMGB1 to elicit immune responses (23). HMGB1-TLR4 stimulates the release of pro-inflammatory cytokines (24). More importantly, combining cDAMPs with PRRs on DCs activates DCs to engulf tumor cells, process tumor antigens, and express these tumor antigens along with MHC-I molecules on the plasma membrane (25). Once activated and maturing, DCs migrate to tumor-draining lymph nodes. Secreted from mature DCs, IL-6, IL-1β, TNF-α, and type γ interferon (IFN-γ) promote the differentiation of T cells into the CD8^+^ phenotype. Then, CD8^+^ T cells are activated by antigen cross-presentation from DCs to become cytotoxic T lymphocytes (CTLs) (20). Ultimately, CTLs induce tumor cell apoptosis through the release of perforin and granzyme B, or a combination of Fas ligand (FasL) with Fas (**Figure 1**) (11, 26).

IR also induces ICD *via* the IFN-I response. Nucleic dsDNA is damaged by IR and released from the nucleus to aggregate in the cytoplasm. Cytosolic dsDNA is sensed by cyclic GMP-AMP (cGAMP) synthase (cGAS), resulting in cGAMP generation. Consequently, the STING–TBK1–IRF3 pathway (STING: stimulator of interferon genes; TBK1: TANK-binding kinase 1; IRF3: interferon regulatory factor 3) is activated, causing IFN-I production and its release from inside to outside the cell (27). The interaction between IFN-I and its receptor on DCs promotes DCs maturation, increases the expression of costimulatory molecules on DCs, and enhances the migration ability of DCs to tumor-draining lymph nodes, eventually promoting antitumor immunity mediated by CD8^+^ T cells (28). IFN-I also stimulates macrophages to secrete inflammatory factors and restrains the immunosuppressive ability of CD4^+^CD25^+^FOXP3^+^ Tregs (29).

## IR-Associated Factors About ICD Activation

IR-driven ICD is not only correlated with tumor genetic background and tumor microenvironment but also linked with post-irradiation time, and the type, dose, and fractionation schedule of radiation (2, 30, 31).

Within a certain range, IR induces ICD in a time-dependent manner. Three radical beams (proton, photon, and carbon ion) drive CRT exposure in four human carcinoma cell lines (CNE-2, A549, U251, and Tca8113), and CRT exposure raises with an increase in post-irradiation time (12, 24, and 48 h) (32). In addition, a study found that the increase in HSP70 and HMGB1 started 24 and 48 h, respectively, in MC-38 cells following irradiation with ^213^Bi (33).

To compare ICD induction by different types of IR (carbon-ion, proton, and photon irradiation), the relative biological effectiveness (RBE) must be taken into consideration. RBE is defined as the ratio of physical doses that lead to the same biological effect (34). The RBE value of proton and photon is regarded as 1.1, but carbon ion is 2–3 (34, 35). Certainly, the RBE value is related with the type of tumor cells. Therefore, the equivalent biological dose (the product of physical dose and RBE) of carbon ion is more than twice that of proton and photon at the same physical dose in the CNE-2 cell line. Yangle Huang et al. confirmed that carbon-ion induced more CRT exposure than proton and photon at low physical dose (2, 4 Gy) due to higher equivalent biological dose of carbon-ion (32). Moreover, the successful activation of ICD requires irradiation dose to be within a precise range; IR doses below or above this threshold produce undesirable immune responses (36). Proton and photon promote CRT exposure in a dose-dependent manner, whereas carbon ion induces more CRT exposure at 4 Gy than 2 and 10 Gy, perhaps owing to the decrease and inhibition of ICD induction by carbon-ion at 2 and 10 Gy separately (32).

IR at different doses produces different types of tumor cell death. Low-dose x-rays (<5 Gy/fraction) activate mitotic catastrophe and apoptosis. High-dose x-rays (8—30 Gy/fraction) induce necrosis and necroptosis (37, 38). Some studies have investigated the optimal dose and fractionation schedule of IR to induce ICD. A study observed that in TSA mammary carcinoma cells, IR enhanced ATP and HMGB1 release and CRT exposure in a dose-dependent manner (2, 5, 10, and 20 Gy) (39). Demaria et al. indicated that single over 5- to 10-Gy x-ray doses were sufficient to produce ICD (40). In glioblastoma cell lines, HSP70 and HMGB1 release are stimulated by the classical fractionation schedule (5×2 Gy) (41). In human prostate cancer cell lines, x-rays induce ATP and HMGB1 secretion at both 1×10 Gy and 10×1 Gy, but the latter elicits more HMGB1 release than the former in DU145 and PC3 cells (42). As mentioned above, dsDNA accumulation in the cytoplasm induces IFN-I release *via* the cGAS–STING pathway. However, Trex1, a DNA exonuclease, can degrade dsDNA. Therefore, RT elicits IFN-I release at 3×8 Gy but inhibits release at 1×20 Gy as a result of the activation of Trex1 (43).

## The Evidence of IR-Induced ICD Biomarkers in the Clinical Setting

In the clinical setting, it is difficult to prove the induction of ICD in patients treated with RT. Some clinical studies combined RT with immunotherapy to verify ICD indirectly according to enhanced systemic tumor responses (2). In fact, RT-driven antitumor immunity is not completely attributed to ICD, as mentioned at the beginning of the review. The current studies might only indirectly verify ICD by detecting cDAMPs in serum or tumor tissue resected from patients after RT (44). One study confirmed that elevated tumor cell surface expression of CRT was detected in tumor samples of patients with renal clear cell carcinoma treated with stereotactic body radiotherapy (SBRT) (45). Takashi Murakami et al. suggested that neoadjuvant chemoradiotherapy induces the overexpression of MHC I-related chain A/B, CRT, and HSP70, and creates a favorable immunogenic tumor microenvironment with a low Treg/tumor infiltrating lymphocyte (TIL) ratio in patients with pancreatic cancer (46). Yoshiyuki Suzuki et al. indicated that chemoradiotherapy induced tumor antigen–specific T-cell responses in patients with esophageal squamous cell carcinoma along with increased HMGB1 release in serum (47).

It has been reported that some cDAMPs might have the potential to predict clinical outcomes of patients following RT (44). For example, HMGB1 was significantly increased in the serum of patients with esophageal cancer after chemoradiation, and its level was positively correlated with patient survival (47, 48). However, cDAMPs as double-edged swords can also promote tumor growth, so elevated cDAMPs expression might be associated with decreased survival (21). In general, whether high HSP70 levels predict positive clinical outcomes depends on its position in cells, tumor types, and its durable upregulation time following RT. It has been confirmed that intracellular HSP70 suppresses tumor cell apoptosis whereas membrane-bound and extracellular HSP70 stimulates the innate and adaptive antitumor immunity to retard tumor growth (49). Stefan Stangl et al. suggested that high intracellular HSP70 expression and low NK cell infiltration in patients with squamous-cell carcinoma of the head and neck were linked with unfavorable outcomes after surgery and radiochemotherapy (RCT) (49). In contrast, progression-free survival and overall survival were significantly better after RCT in patients with primary glioblastomas and having high intracellular HSP70 expression levels (50). Another study demonstrated that the level of free HSP70 was upregulated in the serum of patients with breast cancer up to 6 weeks after RT, which might predict an unfavorable prognosis as a result of a chronic inflammatory response (51).

## Immunogenic Forms of Cell Death

The types of tumor cell death induced by IR include mitotic catastrophe and mitotic death, apoptosis, necrosis, senescence, necroptosis, and ferroptosis (2). Among them, necroptosis and caspase-independent apoptosis elicited by high levels of radiation are regarded as immunogenic forms of cell death (52). A common feature of these cell death types is the release or exposure of cDAMPs; therefore, the latter are the key molecules of initiating antitumor immunity (52). In addition, it is proved that the gold standard for ICD evaluation is to vaccinate immunocompetent mice with dying syngeneic cancer cells and then rechallenge them with live cancer cells. An absence or slowdown of tumor growth would indicate the induction of an adaptive antitumor immune response (53).

As a form of RCD, necroptosis is mediated by receptor-interacting serine/threonine-protein kinase 1 (RIPK1), RIPK3, and mixed lineage kinase domain-like protein (MLKL), and leads to cellular swelling, membrane disruption, and intracellular contents release (54, 55). The successful induction of necroptosis with IR in non-small cell lung cancer (NSCLC) cells depends on irradiation dose, fractionation schedule, and RIPK3 expression. In NSCLC cells with low RIPK3 expression, apoptosis and necroptosis are activated dose-dependently both with <10 Gy/fraction RT and ≥10 Gy/fraction ablative hypofractionated radiation therapy (HFRT). In NSCLC cells with high RIPK3 expression, necroptosis is preferentially stimulated by ≥10 Gy/fraction ablative HFRT. The induction and activation of MLKL and HMGB1 release are involved both *in vitro* and *in vivo* (37). The release of HMGB1 from necroptotic tumor cells that underwent ablative HFRT suggests that necroptosis induced by ablative HFRT is immunogenic and has the potential to activate immune response although there is very little direct evidence available about it. Indeed, necroptosis has been confirmed as ICD in that tumor-bearing mice injected with necroptotic fibroblasts initiate an systemic immune response (56). The activation of the RIPK1/RIPK3/NF-kB pathway in necroptotic cells results in the release of cytokines that promote DC activation and stimulate antitumor immunity mediated by CTLs (56).

Ferroptosis is a form of iron-dependent RCD that is regulated by lipid, iron, and cysteine metabolism with lipid peroxidation and lethal ROS accumulation (10, 57). Like necroptosis, ferroptosis also belongs to regulated necrosis, which can release cDAMPs to activate antitumor immune response (58, 59). A study demonstrated that irradiated tumor cells released microparticles to induce ferroptosis, which promotes cell surface exposure of CRT, ATP secretion, and macrophage phagocytosis (60). Although IR induces ferroptosis indirectly to activate antitumor immune response, there is growing evidence regarding the induction of ferroptosis by IR in tumor cells and the immunogenicity of ferroptosis (42, 61, 62). The connection between IR-induced ferroptosis and antitumor immunity might be linked to many factors such as radiation dose and type. The previous studies have indicated that IR induces tumor cell ferroptosis and ferroptosis improves the radiosensitivity of tumor cells (10, 63, 64). Based on this, RT combined with ferroptosis inducers may improve the therapeutic effect of RT by increasing lipid peroxidation and promoting immune response rather than DNA damage or caspase activation (65). Besides cDAMPs, ferroptotic tumor cells may release oxidized lipids such as arachidonic acid-derived oxidation products and oxidized phospholipids to regulate antitumor immunity, but further studies are needed to confirm this (66).

## The Methods of Enhancing IR-Induced ICD

Many ICD inducers have been developed to promote ICD and augment antitumor immunity, including cytotoxic drugs like anthracyclines or again oncolytic viruses (7). There are some therapies that boost IR-activated ICD, such as nanoparticles, hyperthermia, or chemotherapy. **Table 1** summarizes the methods that further stimulate ICD upon IR.

**Table 1:** Methods promoting RT-induced ICD.

Methods	Tumor cells	ICD hallmarks	References
**Nanoparticles**			
AuNPs	MDA MB 231 cells	CRT exposure	(67)
H@Gd-NCPs	CT26 cells	CRT exposure HMGB1 release ATP secretion	(68)
PLGA-R837@Cat	CT26 cells	CRT exposure	(69)
UCNP-DOX	H460 cells	ATP secretion	(70)
WO2.9-WSe2-PEG	4T1 cells	CRT exposure	(71)
S-AuNC	Tramp C1 cells	CRT exposure HMGB1 release	(72)
**ASTX660**	MOC1 cells	CRT/HSP70 exposure HMGB1 release	(73)
**Hyperthermia**	B16-F10 cells	HSP70/HMGB1 release	(74)
	HCT15 cells	HSP70/HMGB1 release	(75, 76)
	SW480 cells	HMGB1 release	(76)
**Chemotherapy**			
mFX	PDAC cell lines	CRT/ERp57 exposure	(77)
		HMGB1 release	
platinum	TSA cells	CRT exposure HMGB1 release ATP secretion	(39)
**Bip inhibition**	Glioma stem cells	CRT exposure ATP secretion HMGB1 release	(78)

As drug delivery carriers, nanoparticles have high tumor-targeting, permeability and retention effect, and allow to decrease toxicity by reducing drug dosing (79). As a result, the development of nanoparticles has become a research hotspot currently. Nanoparticles are classified into synthetic polymers, biomimetic materials, and inorganic materials according to their physical and chemical properties (80). Some smart nanoparticles are activated by specific conditions such as PH, light, and hyperthermia to release drugs (80). Accumulating studies have combined nanoparticles with immunoadjuvants, chemotherapeutics, photodynamic therapy, or again photothermal therapy in order to reshape the tumor hypoxic microenvironment to elicit or enhance ICD (81, 82). These novel nanoparticles synergized with IR and enhanced antitumor immune function (83–87). Qian Chen et al. confirmed that PLGA-R837@Cat nanoparticle-based x-ray radiation inhibited primary tumors significantly in comparison with RT alone. Moreover, this method generated strong antitumor immune responses with enhanced ICD induction; DC activation and maturation; the secretion of IL-2, IFN-γ, and TNF-α; and CTL infiltration. RT enhanced by PLGA-R837@ Cat combined with anti-CTLA4 checkpoint blockade not only led to complete elimination of primary tumors but also further retarded the growth of metastatic tumors with long-term immune memory protection (69).

Hyperthermia (HT) is a cancer treatment strategy by raising tissue temperature to 39–45°C for a certain period of time and it has been reported in many phase II and phase III trials combined with radiotherapy and chemotherapy (88, 89). HT can not only cause protein denaturation and aggregation to activate downstream pathways such as the DNA damage response to kill tumor cells directly but also induce ICD to reverse tumor immunosuppressive microenvironment and produce antitumor immunity (90). HSPs are the most important cDAMPs to initiate tumor-specific immune responses *via* TLR4 signaling (16, 90). One research treated CT26 tumor cells with RT and/or HT, then injected them subcutaneously into Balb/c, and monitored tumor growth. Cell treatment with RT at 2 or 5 Gy plus HT delayed tumor growth whereas irradiation with 2 Gy alone did not significantly inhibit tumor growth. In an *in vitro* experiment, x-radiation in combination with HT promoted the increase of HSP70 release and the activation, maturation, and cross-presentation of DCs (75). Fractionated RT (3×2 Gy) retards tumor growth significantly in B16-F10 tumor-bearing mice when combined with HT, in comparison with fractionated RT alone. RT with 2 Gy plus HT increases HSP70 and HMGB1 release significantly in tumor cells; RT with 3×2 Gy plus HT facilitates CD8+ T cell, DC, and NK cell infiltration, but inhibits Treg and MDSC infiltration in the tumors (74).

ASTX660 is an inhibitor of apoptosis protein antagonist, resulting in cellular Inhibitor of Apoptosis Proteins 1/2 degradation and X-linked inhibitor of apoptosis protein inhibition (91). Previous research has demonstrated that ASTX660 can potentiate antitumor immunity by enhancing the sensitivity of tumor cells to induction of TNFR superfamily ligation-related apoptosis downstream (92). The combination of ASTX660 and RT significantly boosts tumor regression through the function of TNFα, CD8^+^ T cells, and NK cells (92). Wenda Ye et al. indicated that ASTX660 further facilitated CRT and HSP70 exposure and HMGB1 release in MOC1 cells when combined with IR as compared to ASTX660 alone. In *in vivo* experiments, ASTX660 heightened IR-induced ICD modestly to inhibit tumor generation in 72% of mice in contrast with 50% of mice treated with IR alone. In addition, ASTX660 combined with IR promoted clonal expansion of TIL by enhancing DCs activation and MHC I expression (73).

As a traditional therapy, chemotherapy is widely used in advanced cancer treatment and functions as the auxiliary therapy following surgery. Consistent with RT, chemotherapy induces ICD characterized by CRT exposure, ATP, and HMGB1 secretion through collateral ER stress effects (93). Nevertheless, not all chemotherapeutics can activate ICD, and chemotherapeutics certified to have the ability of ICD induction include idarubicin, epirubicin, doxorubicin, mitoxantrone, oxaliplatin, bortezomib, and cyclophosphamide (94). Concurrent treatment with a modified dose of FOLFIRINOX (mFX) and SBRT enhanced antitumor efficacy and produced long-term systemic antitumor immunity. KCKO cells treated with mFX and SBRT were subcutaneously injected into mice. The results showed that percentage of tumor-free mice was 100% and enhanced ICD was verified in tumor tissue featured by increased HMGB1 release and CRT exposure compared with single treatment. Treatment with mFX and SBRT boosted the phagocytosis, maturation, and cross-presentation of DCs and intratumoral infiltration of CD8^+^ T cells (77).

Known as a molecular chaperone in the ER, binding immunoglobulin protein (Bip) plays a key role in the unfolded protein response to protect cells from denatured proteins and to suppress apoptosis (95). It has also been reported that Bip expression is associated with increased tumor progression (96). Accordingly, Bip inhibition can inhibit tumor growth by enhancing ER stress to elicit cDAMPs release or exposure to activate antitumor immunity (78). The combination of Bip inhibition and 10 Gy IR enhanced IR-induced ICD with increased CRT exposure, ATP secretion, and HMGB1 release and promoted the phagocytosis and maturation of DCs. As a vaccine, glioma stem cells treated with Bip inhibition and IR could delay tumor generation efficiently, facilitate the proliferation of CD4^+^ and CD8^+^ T cells, and decrease Treg cell infiltration *in vivo* (78).

## Conclusion and Perspectives

IR can induce ICD in tumor cells to produce an adaptive immune response mediated by CD8^+^ T cells. The irradiation dosage and fractionation schedule for ICD induction have not been determined, so future research should focus on proper dose and fractionation to activate ICD efficiently, while keeping side effects tolerable. Currently, there are few clinical studies on IR-induced ICD and ICD verification remains indirect. Thus, the number and function of immune cells activated by cDAMP should be further detected. In addition, necroptosis and ferroptosis have dual effects for regulating immune response; thus, it is pivotal to induce these cell death forms to a beneficial direction for cancer treatment.

## Author Contributions

MZ wrote and edited the manuscript. MY, JZ, SQ, HZ, and FY participated in the conception and design of the study. YY, XF, and YZ collected the literature and data. MY, JZ, SQ, HZ, and FY modified grammatical errors and provided intellectual guidance. All authors contributed to the article and approved the submitted version.

## Funding

National Natural Science Foundation of China, Grant/Award Number: No.82071956; Clinical Research Plan of SHDC, Grant/Award Number: No.2020CR4065.

## Conflict of Interest

The authors declare that the research was conducted in the absence of any commercial or financial relationships that could be construed as a potential conflict of interest.

## Publisher’s Note

All claims expressed in this article are solely those of the authors and do not necessarily represent those of their affiliated organizations, or those of the publisher, the editors and the reviewers. Any product that may be evaluated in this article, or claim that may be made by its manufacturer, is not guaranteed or endorsed by the publisher.

## Abbreviations

ASC, Apoptosis-associated speck-like protein containing a caspase recruit domain; ATP, Adenosine triphosphate; Bip, Binding immunoglobulin protein; cDAMPs, Constitutive damage-associated molecular patterns; cGAMP, Cyclic GMP-AMP; cGAS, Cyclic GMP-AMP synthase; CRT, Calreticulin; CTL, Cytotoxic T lymphocyte; CXCL, C-X-C motif chemokine ligand; DAMPs, Damage-associated molecular patterns; DC, Dendritic cell; dsDNA, Double-strand DNA; ER, Endoplasmic reticulum; Fas, Factor related apoptosis; FasL, Factor related apoptosis ligand; HFRT, Hypofractionated radiation therapy; HMGB1, High mobility group box 1; HSPs, Heat shock proteins; HT, Hyperthermia; ICD, Immunogenic cell death; iDAMPs, Inducible damage-associated molecular patterns; IFN-I, Type I interferon; IFN-γ, Type γ interferon; IL-1β, Interleukin-1β; IL-6, Interleukin-6; IR, Ionizing radiation; IRF3, Interferon regulatory factor 3; mFX, Modified dose of FOLFIRINOX; MHC I, Major histocompatibility complex class I; MLKL, Mixed lineage kinase domain-like protein; NLRP3, NOD-like receptor protein 3; NSCLC, Non-small cell lung cancer; P2RX7, P2X purinoceptor 7; P2RY2, Purinergic receptor P2Y2; RBE, Relative biological effectiveness; RCD, Regulated cell death; RCT, Radiochemotherapy; RIPK1, Receptor-interacting serine/threonine-protein kinase 1; ROS, Reactive oxygen species; RT, Radiotherapy; SBRT, Stereotactic body radiotherapy; STING, Stimulator of interferon genes; TBK1, TANK-binding kinase 1; TCR, T-cell receptor; TIL, Tumor-infiltrating lymphocyte; TLR4, Toll-like receptor 4; TNF-α, Tumor necrosis factor-α.

## References

[1] Rodriguez-Ruiz ME Vitale I Harrington KJ Melero I Galluzzi L. Immunological Impact of Cell Death Signaling Driven by Radiation on the Tumor Microenvironment. Nat Immunol (2020) 21:120–34. 10.1038/s41590-019-0561-4 31873291

[2] Sia J Szmyd R Hau E Gee HE. Molecular Mechanisms of Radiation-Induced Cancer Cell Death: A Primer. Front Cell Dev Biol (2020) 8:41. 10.3389/fcell.2020.00041 32117972 PMC7031160

[3] Welsh W Bevelacqua JJ Dobrzyński L Mortazavi SAR Farjadian SH Mortazavi SMJ. Abscopal Effect Following Radiation Therapy in Cancer Patients: A New Look From the Immunological Point of View. J BioMed Phys Eng (2020) 10:537–42. 10.31661/jbpe.v0i0.1066 PMC7416099 32802801

[4] Yoo GS Ahn WG Kim SY Kang W Choi C Park HC. Radiation-Induced Abscopal Effect and Its Enhancement by Programmed Cell Death 1 Blockade in the Hepatocellular Carcinoma: A Murine Model Study. Clin Mol Hepatol (2021) 27:144–56. 10.3350/cmh.2020.0095 PMC7820196 33280350

[5] Sharabi AB Lim M DeWeese TL Drake CG. Radiation and Checkpoint Blockade Immunotherapy: Radiosensitisation and Potential Mechanisms of Synergy. Lancet Oncol (2015) 16:e498–509. 10.1016/S1470-2045(15)00007-8 26433823

[6] Rapoport BL Anderson R. Realizing the Clinical Potential of Immunogenic Cell Death in Cancer Chemotherapy and Radiotherapy. Int J Mol Sci (2019) 20:959. 10.3390/ijms20040959 PMC6412296 30813267

[7] Li X. The Inducers of Immunogenic Cell Death for Tumor Immunotherapy. Tumori (2018) 104:1–8. 10.5301/tj.5000675 28967094

[8] Galluzzi L Vitale I Warren S Adjemian S Agostinis P Martinez AB. Consensus Guidelines for the Definition, Detection and Interpretation of Immunogenic Cell Death. J Immunother Cancer (2020) 8:e000337. 10.1136/jitc-2019-000337 32209603 PMC7064135

[9] Nehs MA Lin CI Kozono DE Whang EE Cho NL Zhu K. Necroptosis Is a Novel Mechanism of Radiation-Induced Cell Death in Anaplastic Thyroid and Adrenocortical Cancers. Surgery (2011) 150:1032–9. 10.1016/j.surg.2011.09.012 22136818

[10] Lang X Green MD Wang W Yu J Choi JE Jiang L. Radiotherapy and Immunotherapy Promote Tumoral Lipid Oxidation and Ferroptosis via Synergistic Repression of SLC7A11. Cancer Discov (2019) 9:1673–85. 10.1158/2159-8290.CD-19-0338 PMC6891128 31554642

[11] Aaes TL Vandenabeele P. The Intrinsic Immunogenic Properties of Cancer Cell Lines, Immunogenic Cell Death, and How These Influence Host Antitumor Immune Responses. Cell Death Differ (2021) 28:843–60. 10.1038/s41418-020-00658-y PMC7937679 33214663

[12] Radogna F Diederich M. Stress-Induced Cellular Responses in Immunogenic Cell Death: Implications for Cancer Immunotherapy. Biochem Pharmacol (2018) 153:12–23. 10.1016/j.bcp.2018.02.006 29438676

[13] Bezu L Sauvat A Humeau J Gomes-da-Silva LC Iribarren K Forveille S. Eif2α Phosphorylation Is Pathognomonic for Immunogenic Cell Death. Cell Death Differ (2018) 25:1375–93. 10.1038/s41418-017-0044-9 PMC6113215 29358668

[14] Zhou J Wang G Chen Y Wang H Hua Y Cai Z. Immunogenic Cell Death in Cancer Therapy: Present and Emerging Inducers. J Cell Mol Med (2019) 23:4854–65. 10.1111/jcmm.14356 PMC6653385 31210425

[15] Garg AD Agostinis P. Cell Death and Immunity in Cancer: From Danger Signals to Mimicry of Pathogen Defense Responses. Immunol Rev (2017) 280:126–48. 10.1111/imr.12574 29027218

[16] Adkins I Fucikova J Garg AD Agostinis P Spisek R. Physical Modalities Inducing Immunogenic Tumor Cell Death for Cancer Immunotherapy. Oncoimmunology (2014) 3:e968434. 10.4161/21624011.2014.968434 25964865 PMC4352954

[17] Young CNJ Gorecki DC. P2RX7 Purinoceptor as a Therapeutic Target-The Second Coming? Front Chem (2018) 6:248. 10.3389/fchem.2018.00248 30003075 PMC6032550

[18] Keam S Gill S Ebert MA Nowak AK Cook AM. Enhancing the Efficacy of Immunotherapy Using Radiotherapy. Clin Transl Immunol (2020) 9:e1169. 10.1002/cti2.1169 PMC7507442 32994997

[19] Xing P Liao Z Ren Z Zhao J Song F Wang G. Roles of Low-Density Lipoprotein Receptor-Related Protein 1 in Tumors. Chin J Cancer (2016) 35:6. 10.1186/s40880-015-0064-0 26738504 PMC4704379

[20] Kielbik M Szulc-Kielbik I Klink M. Calreticulin-Multifunctional Chaperone in Immunogenic Cell Death: Potential Significance as a Prognostic Biomarker in Ovarian Cancer Patients. Cells (2021) 10:130. 10.3390/cells10010130 33440842 PMC7827772

[21] Ashrafizadeh M Farhood B Eleojo Musa A Taeb S Najafi M. Damage-Associated Molecular Patterns in Tumor Radiotherapy. Int Immunopharmacol (2020) 86:106761. 10.1016/j.intimp.2020.106761 32629409

[22] Djureinovic D Wang M Kluger HM. Agonistic CD40 Antibodies in Cancer Treatment. Cancers (Basel) (2021) 13:1302. 10.3390/cancers13061302 33804039 PMC8000216

[23] Kashani B Zandi Z Pourbagheri-Sigaroodi A Bashash D Ghaffari SH. The Role of Toll-Like Receptor 4 (TLR4) in Cancer Progression: A Possible Therapeutic Target? J Cell Physiol (2021) 236:4121–37. 10.1002/jcp.30166 33230811

[24] Liao Y Liu S Fu S Wu J. HMGB1 in Radiotherapy: A Two Headed Signal Regulating Tumor Radiosensitivity and Immunity. Onco Targets Ther (2020) 13:6859–71. 10.2147/OTT.S253772 PMC7369309 32764978

[25] Ma Y Pitt JM Li Q Yang H. The Renaissance of Anti-Neoplastic Immunity From Tumor Cell Demise. Immunol Rev (2017) 280:194–206. 10.1111/imr.12586 29027231

[26] Ahmed A Tait SWG. Targeting Immunogenic Cell Death in Cancer. Mol Oncol (2020) 14:2994–3006. 10.1002/1878-0261.12851 33179413 PMC7718954

[27] Kho VM Mekers VE Span PN Bussink J Adema GJ. Radiotherapy and cGAS/STING Signaling: Impact on MDSCs in the Tumor Microenvironment. Cell Immunol (2021) 362:104298. 10.1016/j.cellimm.2021.104298 33592541

[28] Storozynsky Q Hitt MM. The Impact of Radiation-Induced DNA Damage on cGAS-STING-Mediated Immune Responses to Cancer. Int J Mol Sci (2020) 21:8877. 10.3390/ijms21228877 PMC7700321 33238631

[29] Fucikova J Kepp O Kasikova L Petroni G Yamazaki T Liu P. Detection of Immunogenic Cell Death and Its Relevance for Cancer Therapy. Cell Death Dis (2020) 11:1013. 10.1038/s41419-020-03221-2 33243969 PMC7691519

[30] Li H Jin X Chen B Li P Li Q. Autophagy-Regulating microRNAs: Potential Targets for Improving Radiotherapy. J Cancer Res Clin Oncol (2018) 144:1623–34. 10.1007/s00432-018-2675-8 PMC11813381 29971533

[31] Ko A Kanehisa A Martins I Senovilla L Chargari C Dugue D. Autophagy Inhibition Radiosensitizes *In Vitro*, Yet Reduces Radioresponses *In Vivo* Due to Deficient Immunogenic Signalling. Cell Death Differ (2014) 21:92–9. 10.1038/cdd.2013.124 PMC3857616 24037090

[32] Huang Y Dong Y Zhao J Zhang L Kong L Lu JJ. Comparison of the Effects of Photon, Proton and Carbon-Ion Radiation on the Ecto-Calreticulin Exposure in Various Tumor Cell Lines. Ann Transl Med (2019) 7:542. 10.21037/atm.2019.09.128 31807524 PMC6861809

[33] Gorin JB Menager J Gouard S Maurel C Guilloux Y Faivre-Chauvet A. Antitumor Immunity Induced After Alpha Irradiation. Neoplasia (2014) 16:319–28. 10.1016/j.neo.2014.04.002 PMC4094834 24862758

[34] Beltran C Schultz HL Anand A Merrell K. Radiation Biology Considerations of Proton Therapy for Gastrointestinal Cancers. J Gastrointest Oncol (2020) 11:225–30. 10.21037/jgo.2019.06.08 PMC7052770 32175125

[35] Lehrer EJ Prabhu AV Sindhu KK Lazarev S Ruiz-Garcia H Peterson JL. Proton and Heavy Particle Intracranial Radiosurgery. Biomedicines (2021) 9:31. 10.3390/biomedicines9010031 33401613 PMC7823941

[36] Bernstein MB Krishnan S Hodge JW Chang JY. Immunotherapy and Stereotactic Ablative Radiotherapy (ISABR): A Curative Approach? Nat Rev Clin Oncol (2016) 13:516–24. 10.1038/nrclinonc.2016.30 PMC6053911 26951040

[37] Wang HH Wu ZQ Qian D Zaorsky NG Qiu MH Cheng JJ. Ablative Hypofractionated Radiation Therapy Enhances Non-Small Cell Lung Cancer Cell Killing via Preferential Stimulation of Necroptosis *In Vitro* and *In Vivo* . Int J Radiat Oncol Biol Phys (2018) 101:49–62. 10.1016/j.ijrobp.2018.01.036 29619976

[38] Kabiljo J Harpain F Carotta S Bergmann M. Radiotherapy as a Backbone for Novel Concepts in Cancer Immunotherapy. Cancers (Basel) (2019) 12:79. 10.3390/cancers12010079 PMC7017108 31905723

[39] Golden EB Frances D Pellicciotta I Demaria S Helen Barcellos-Hoff M Formenti SC. Radiation Fosters Dose-Dependent and Chemotherapy-Induced Immunogenic Cell Death. Oncoimmunology (2014) 3:e28518. 10.4161/onci.28518 25071979 PMC4106151

[40] Vandenabeele P Vandecasteele K Bachert C Krysko O Krysko DV. Immunogenic Apoptotic Cell Death and Anticancer Immunity. Adv Exp Med Biol (2016) 930:133–49. 10.1007/978-3-319-39406-0_6 27558820

[41] Rubner Y Muth C Strnad A Derer A Sieber R Buslei R. Fractionated Radiotherapy Is the Main Stimulus for the Induction of Cell Death and of Hsp70 Release of P53 Mutated Glioblastoma Cell Lines. Radiat Oncol (2014) 9:89. 10.1186/1748-717X-9-89 24678590 PMC3994240

[42] Aryankalayil MJ Makinde AY Gameiro SR Hodge JW Rivera-Solis PP Palayoor ST. Defining Molecular Signature of Pro-Immunogenic Radiotherapy Targets in Human Prostate Cancer Cells. Radiat Res (2014) 182:139–48. 10.1667/RR13731.1 PMC4216662 25003313

[43] Sridharan V Schoenfeld JD. Immunotherapy and Radiation: Charting a Path Forward Together. Hematol Oncol Clin North Am (2019) 33:1057–69. 10.1016/j.hoc.2019.08.001 31668206

[44] Vaes RDW Hendriks LEL Vooijs M De Ruysscher D. Biomarkers of Radiotherapy-Induced Immunogenic Cell Death. Cells (2021) 10:930. 10.3390/cells10040930 33920544 PMC8073519

[45] Singh AK Winslow TB Kermany MH Goritz V Heit L Miller A. A Pilot Study of Stereotactic Body Radiation Therapy Combined With Cytoreductive Nephrectomy for Metastatic Renal Cell Carcinoma. Clin Cancer Res (2017) 23:5055–65. 10.1158/1078-0432.CCR-16-2946 PMC5581708 28630212

[46] Murakami T Homma Y Matsuyama R Mori R Miyake K Tanaka Y. Neoadjuvant Chemoradiotherapy of Pancreatic Cancer Induces a Favorable Immunogenic Tumor Microenvironment Associated With Increased Major Histocompatibility Complex Class I-Related Chain A/B Expression. J Surg Oncol (2017) 116:416–26. 10.1002/jso.24681 28608409

[47] Suzuki Y Mimura K Yoshimoto Y Watanabe M Ohkubo Y Izawa S. Immunogenic Tumor Cell Death Induced by Chemoradiotherapy in Patients With Esophageal Squamous Cell Carcinoma. Cancer Res (2012) 72:3967–76. 10.1158/0008-5472.CAN-12-0851 22700877

[48] Huang CY Chiang SF Ke TW Chen TW Lan YC You YS. Cytosolic High-Mobility Group Box Protein 1 (HMGB1) and/or PD-1+ TILs in the Tumor Microenvironment May Be Contributing Prognostic Biomarkers for Patients With Locally Advanced Rectal Cancer Who Have Undergone Neoadjuvant Chemoradiotherapy. Cancer Immunol Immunother (2018) 67:551–62. 10.1007/s00262-017-2109-5 PMC11028045 29270668

[49] Stangl S Tontcheva N Sievert W Shevtsov M Niu M Schmid TE. Heat Shock Protein 70 and Tumor-Infiltrating NK Cells as Prognostic Indicators for Patients With Squamous Cell Carcinoma of the Head and Neck After Radiochemotherapy: A Multicentre Retrospective Study of the German Cancer Consortium Radiation Oncology Group (DKTK-ROG). Int J Cancer (2018) 142:1911–25. 10.1002/ijc.31213 PMC5873418 29235112

[50] Lammer F Delbridge C Wurstle S Neff F Meyer B Schlegel J. Cytosolic Hsp70 as a Biomarker to Predict Clinical Outcome in Patients With Glioblastoma. PloS One (2019) 14:e0221502. 10.1371/journal.pone.0221502 31430337 PMC6701831

[51] Rothammer A Sage EK Werner C Combs SE Multhoff G. Increased Heat Shock Protein 70 (Hsp70) Serum Levels and Low NK Cell Counts After Radiotherapy - Potential Markers for Predicting Breast Cancer Recurrence? Radiat Oncol (2019) 14:78. 10.1186/s13014-019-1286-0 31077235 PMC6509784

[52] van Schaik TA Chen KS Shah K. Therapy-Induced Tumor Cell Death: Friend or Foe of Immunotherapy? Front Oncol (2021) 11:678562. 10.3389/fonc.2021.678562 34141622 PMC8204251

[53] Tang R Xu J Zhang B Liu J Liang C Hua J. Ferroptosis, Necroptosis, and Pyroptosis in Anticancer Immunity. J Hematol Oncol (2020) 13:110. 10.1186/s13045-020-00946-7 32778143 PMC7418434

[54] Woo Y Lee HJ Jung YM Jung YJ. Regulated Necrotic Cell Death in Alternative Tumor Therapeutic Strategies. Cells (2020) 9:2709. 10.3390/cells9122709 PMC7767016 33348858

[55] Krysko O Aaes TL Kagan VE D'Herde K Bachert C Leybaert L. Necroptotic Cell Death in Anti-Cancer Therapy. Immunol Rev (2017) 280:207–19. 10.1111/imr.12583 29027225

[56] Snyder AG Hubbard NW Messmer MN Kofman SB Hagan CE Orozco SL. Intratumoral Activation of the Necroptotic Pathway Components RIPK1 and RIPK3 Potentiates Antitumor Immunity. Sci Immunol (2019) 4:eaaw2004. 10.1126/sciimmunol.aaw2004 31227597 PMC6831211

[57] Zhang Z Lu M Chen C Tong X Li Y Yang K. Holo-Lactoferrin: The Link Between Ferroptosis and Radiotherapy in Triple-Negative Breast Cancer. Theranostics (2021) 11:3167–82. 10.7150/thno.52028 PMC7847686 33537080

[58] Garg AD Romano E Rufo N Agostinis P. Immunogenic Versus Tolerogenic Phagocytosis During Anticancer Therapy: Mechanisms and Clinical Translation. Cell Death Differ (2016) 23:938–51. 10.1038/cdd.2016.5 PMC4987738 26891691

[59] Tang D Kepp O Kroemer G. Ferroptosis Becomes Immunogenic: Implications for Anticancer Treatments. Oncoimmunology (2020) 10:1862949. 10.1080/2162402X.2020.1862949 33457081 PMC7781761

[60] Wan C Sun Y Tian Y Lu L Dai X Meng J. Irradiated Tumor Cell-Derived Microparticles Mediate Tumor Eradication via Cell Killing and Immune Reprogramming. Sci Adv (2020) 6:eaay9789. 10.1126/sciadv.aay9789 32232155 PMC7096163

[61] Lei G Zhang Y Koppula P Liu X Zhang J Lin SH. The Role of Ferroptosis in Ionizing Radiation-Induced Cell Death and Tumor Suppression. Cell Res (2020) 30:146–62. 10.1038/s41422-019-0263-3 PMC7015061 31949285

[62] Chen X Kang R Kroemer G Tang D. Broadening Horizons: The Role of Ferroptosis in Cancer. Nat Rev Clin Oncol (2021) 18:280–96. 10.1038/s41571-020-00462-0 33514910

[63] Wang W Green M Choi JE Gijon M Kennedy PD Johnson JK. CD8(+) T Cells Regulate Tumour Ferroptosis During Cancer Immunotherapy. Nature (2019) 569:270–4. 10.1038/s41586-019-1170-y PMC6533917 31043744

[64] Yuan Y Cao W Zhou H Qian H Wang H. CLTRN, Regulated by NRF1/RAN/DLD Protein Complex, Enhances Radiation Sensitivity of Hepatocellular Carcinoma Cells Through Ferroptosis Pathway. Int J Radiat Oncol Biol Phys (2021) 110:859–71. 10.1016/j.ijrobp.2020.12.062 33508374

[65] Ye LF Chaudhary KR Zandkarimi F Harken AD Kinslow CJ Upadhyayula PS. Radiation-Induced Lipid Peroxidation Triggers Ferroptosis and Synergizes With Ferroptosis Inducers. ACS Chem Biol (2020) 15:469–84. 10.1021/acschembio.9b00939 PMC7180072 31899616

[66] Friedmann Angeli JP Krysko DV Conrad M. Ferroptosis at the Crossroads of Cancer-Acquired Drug Resistance and Immune Evasion. Nat Rev Cancer (2019) 19:405–14. 10.1038/s41568-019-0149-1 31101865

[67] Janic B Brown SL Neff R Liu F Mao G Chen Y. Therapeutic Enhancement of Radiation and Immunomodulation by Gold Nanoparticles in Triple Negative Breast Cancer. Cancer Biol Ther (2021) 22:124–35. 10.1080/15384047.2020.1861923 PMC7928016 33459132

[68] Huang Z Wang Y Yao D Wu J Hu Y Yuan A. Nanoscale Coordination Polymers Induce Immunogenic Cell Death by Amplifying Radiation Therapy Mediated Oxidative Stress. Nat Commun (2021) 12:145. 10.1038/s41467-020-20243-8 33420008 PMC7794559

[69] Chen Q Chen J Yang Z Xu J Xu L Liang C. Nanoparticle-Enhanced Radiotherapy to Trigger Robust Cancer Immunotherapy. Adv Mater (2019) 31:e1802228. 10.1002/adma.201802228 30663118

[70] Qin X Liu J Xu Y Li B Cheng J Wu X. Mesoporous Bi-Containing Radiosensitizer Loading With DOX to Repolarize Tumor-Associated Macrophages and Elicit Immunogenic Tumor Cell Death to Inhibit Tumor Progression. ACS Appl Mater Interfaces (2020) 12:31225–34. 10.1021/acsami.0c08074 32551494

[71] Dong X Cheng R Zhu S Liu H Zhou R Zhang C. A Heterojunction Structured WO2.9-WSe2 Nanoradiosensitizer Increases Local Tumor Ablation and Checkpoint Blockade Immunotherapy Upon Low Radiation Dose. ACS Nano (2020) 14:5400–16. 10.1021/acsnano.9b08962 32324373

[72] Choi B Choi H Yu B Kim DH. Synergistic Local Combination of Radiation and Anti-Programmed Death Ligand 1 Immunotherapy Using Radiation-Responsive Splintery Metallic Nanocarriers. ACS Nano (2020) 14:13115–26. 10.1021/acsnano.0c04701 32885958

[73] Ye W Gunti S Allen CT Hong Y Clavijo PE Van Waes C. ASTX660, an Antagonist of Ciap1/2 and XIAP, Increases Antigen Processing Machinery and Can Enhance Radiation-Induced Immunogenic Cell Death in Preclinical Models of Head and Neck Cancer. Oncoimmunology (2020) 9:1710398. 10.1080/2162402X.2019.1710398 32002309 PMC6959437

[74] Werthmoller N Frey B Ruckert M Lotter M Fietkau R Gaipl US. Combination of Ionising Radiation With Hyperthermia Increases the Immunogenic Potential of B16-F10 Melanoma Cells *In Vitro* and *In Vivo* . Int J Hyperthermia (2016) 32:23–30. 10.3109/02656736.2015.1106011 26754406

[75] Schildkopf P Frey B Ott OJ Rubner Y Multhoff G Sauer R. Radiation Combined With Hyperthermia Induces HSP70-Dependent Maturation of Dendritic Cells and Release of Pro-Inflammatory Cytokines by Dendritic Cells and Macrophages. Radiother Oncol (2011) 101:109–15. 10.1016/j.radonc.2011.05.056 21704416

[76] Schildkopf P Frey B Mantel F Ott OJ Weiss EM Sieber R. Application of Hyperthermia in Addition to Ionizing Irradiation Fosters Necrotic Cell Death and HMGB1 Release of Colorectal Tumor Cells. Biochem Biophys Res Commun (2010) 391:1014–20. 10.1016/j.bbrc.2009.12.008 19968962

[77] Ye J Mills BN Zhao T Han BJ Murphy JD Patel AP. Assessing the Magnitude of Immunogenic Cell Death Following Chemotherapy and Irradiation Reveals a New Strategy to Treat Pancreatic Cancer. Cancer Immunol Res (2020) 8:94–107. 10.1158/2326-6066.CIR-19-0373 31719057 PMC6946873

[78] Yang W Xiu Z He Y Huang W Li Y Sun T. Bip Inhibition in Glioma Stem Cells Promotes Radiation-Induced Immunogenic Cell Death. Cell Death Dis (2020) 11:786. 10.1038/s41419-020-03000-z 32963254 PMC7508950

[79] Gao J Wang WQ Pei Q Lord MS Yu HJ. Engineering Nanomedicines Through Boosting Immunogenic Cell Death for Improved Cancer Immunotherapy. Acta Pharmacol Sin (2020) 41:986–94. 10.1038/s41401-020-0400-z PMC7470797 32317755

[80] Qi J Jin F Xu X Du Y. Combination Cancer Immunotherapy of Nanoparticle-Based Immunogenic Cell Death Inducers and Immune Checkpoint Inhibitors. Int J Nanomedicine (2021) 16:1435–56. 10.2147/IJN.S285999 PMC7910111 33654395

[81] Lim S Park J Shim MK Um W Yoon HY Ryu JH. Recent Advances and Challenges of Repurposing Nanoparticle-Based Drug Delivery Systems to Enhance Cancer Immunotherapy. Theranostics (2019) 9:7906–23. 10.7150/thno.38425 PMC6831456 31695807

[82] Duan X Chan C Lin W. Nanoparticle-Mediated Immunogenic Cell Death Enables and Potentiates Cancer Immunotherapy. Angew Chem Int Ed Engl (2019) 58:670–80. 10.1002/anie.201804882 PMC7837455 30016571

[83] Ding B Zheng P Jiang F Zhao Y Wang M Chang M. MnOx Nanospikes as Nanoadjuvants and Immunogenic Cell Death Drugs With Enhanced Antitumor Immunity and Antimetastatic Effect. Angew Chem Int Ed Engl (2020) 59:16381–4. 10.1002/anie.202005111 32484598

[84] Sen S Hufnagel S Maier EY Aguilar I Selvakumar J DeVore JE. Rationally Designed Redox-Active Au(I) N-Heterocyclic Carbene: An Immunogenic Cell Death Inducer. J Am Chem Soc (2020) 142:20536–41. 10.1021/jacs.0c09753 PMC9398512 33237764

[85] Chattopadhyay S Liu YH Fang ZS Lin CL Lin JC Yao BY. Synthetic Immunogenic Cell Death Mediated by Intracellular Delivery of STING Agonist Nanoshells Enhances Anticancer Chemo-Immunotherapy. Nano Lett (2020) 20:2246–56. 10.1021/acs.nanolett.9b04094 32160474

[86] Wang L Guan R Xie L Liao X Xiong K Rees TW. An ER-Targeting Iridium(III) Complex That Induces Immunogenic Cell Death in Non-Small-Cell Lung Cancer. Angew Chem Int Ed Engl (2021) 60:4657–65. 10.1002/anie.202013987 33217194

[87] Mishchenko T Mitroshina E Balalaeva I Krysko O Vedunova M Krysko DV. An Emerging Role for Nanomaterials in Increasing Immunogenicity of Cancer Cell Death. Biochim Biophys Acta Rev Cancer (2019) 1871:99–108. 10.1016/j.bbcan.2018.11.004 30528646

[88] Stephen ZR Zhang M. Recent Progress in the Synergistic Combination of Nanoparticle-Mediated Hyperthermia and Immunotherapy for Treatment of Cancer. Adv Healthc Mater (2021) 10:e2001415. 10.1002/adhm.202001415 33236511 PMC8034553

[89] Li Z Deng J Sun J Ma Y. Hyperthermia Targeting the Tumor Microenvironment Facilitates Immune Checkpoint Inhibitors. Front Immunol (2020) 11:595207. 10.3389/fimmu.2020.595207 33240283 PMC7680736

[90] Chang M Hou Z Wang M Li C Lin J. Recent Advances in Hyperthermia Therapy-Based Synergistic Immunotherapy. Adv Mater (2021) 33:e2004788. 10.1002/adma.202004788 33289219

[91] Ward GA Lewis EJ Ahn JS Johnson CN Lyons JF Martins V. ASTX660, a Novel Non-Peptidomimetic Antagonist of Ciap1/2 and XIAP, Potently Induces TNFalpha-Dependent Apoptosis in Cancer Cell Lines and Inhibits Tumor Growth. Mol Cancer Ther (2018) 17:1381–91. 10.1158/1535-7163.MCT-17-0848 29695633

[92] Xiao R Allen CT Tran L Patel P Park SJ Chen Z. Antagonist of Ciap1/2 and XIAP Enhances Anti-Tumor Immunity When Combined With Radiation and PD-1 Blockade in a Syngeneic Model of Head and Neck Cancer. Oncoimmunology (2018) 7:e1471440. 10.1080/2162402X.2018.1471440 30393585 PMC6209421

[93] Wang Q Ju X Wang J Fan Y Ren M Zhang H. Immunogenic Cell Death in Anticancer Chemotherapy and Its Impact on Clinical Studies. Cancer Lett (2018) 438:17–23. 10.1016/j.canlet.2018.08.028 30217563

[94] Vanmeerbeek I Sprooten J De Ruysscher D Tejpar S Vandenberghe P Fucikova J. Trial Watch: Chemotherapy-Induced Immunogenic Cell Death in Immuno-Oncology. Oncoimmunology (2020) 9:1703449. 10.1080/2162402X.2019.1703449 32002302 PMC6959434

[95] Dauer P Sharma NS Gupta VK Durden B Hadad R Banerjee S. ER Stress Sensor, Glucose Regulatory Protein 78 (GRP78) Regulates Redox Status in Pancreatic Cancer Thereby Maintaining "Stemness". Cell Death Dis (2019) 10:132. 10.1038/s41419-019-1408-5 30755605 PMC6372649

[96] Kashkoulinejad-Kouhi T Safarian S Arnaiz B Saa L. Enhancement of Cisplatin Sensitivity in Human Breast Cancer MCF-7 Cell Line Through BiP and 14-3-3zeta Co-Knockdown. Oncol Rep (2020) 45:665–79. 10.3892/or.2020.7898 PMC7757084 33416155

